# Supplementary material for: Diagnostic performance of four lateral flow immunoassays for COVID-19 antibodies in Peruvian population
Source: PLOS Glob Public Health. 2023 Jun 2;3(6):e0001555. doi: 10.1371/journal.pgph.0001555 (PMC10237462; doi:10.1371/journal.pgph.0001555)
Supplement: Attachment — Submitted filename: Response to reviewers.docx [file pgph.0001555.s001.docx]

Lima - Peru, March 18, 2023

Miguel Angel Garcia-Bereguiain, PhD

Academic Editor

PLOS Global Public Health

Subject: Submission of revised paper "PGPH-D-22-02126": Diagnostic performance of lateral flow immunoassays for COVID-19 antibodies in Peruvian population

Dear Academic Editor Dr. Garcia-Bereguiain:

Thank you for your email enclosing the reviewers’ comments. We have carefully reviewed the comments and have revised the manuscript accordingly. Our responses are given in a point-by-point manner below. Additionally, please find attached a revised version of our manuscript with tracked changes.

We hope the revised version is now suitable for publication, please do not hesitate to contact us if something else is needed from our behalf, we look forward to hearing from you.

Sincerely,

Dr. Cesar Ugarte-Gil

Assistant Professor

Universidad Peruana Cayetano Heredia

**Response to Reviewer 1:**

Thank you for your review of our paper. We have answered each of your points below.

**1. Correct the extra space in the word SARS-CoV-2 located in the Background section, add “and” in the numbering of study design cassette brands, add the acronym after Universidad Peruana Cayetano Heredia in Lima, Peru: UPCH.**

Regarding the first observation, there are four times in which the word SARS-CoV-2 is written in the background section, but we could not find the extra space you mentioned. Regarding the remaining observations, we have added the "and" (p. 5, line 133) in the numbering of study design cassette brands, and the acronym "UPCH" as suggested (p. 5, line 139).

**2. Place in the abstract the brands.**

Brands have been added to the abstract as indicated (p. 2, lines 40-41).

**3. Consider the change in the name of the article, specifying the number of brands evaluated.**

Title has been changed with the number of brands specified in it as suggested.

**4. Table 1 can be deleted, just emphasize in the text that precedes it.**

Table 1 has been deleted as suggested and Table 2 has now been changed to Table 1.

**5. On page 6, add the references of the manuals corresponding to the evaluated brands.**

The references for the brands evaluated and their IFU/manuals have been added as indicated (p. 5, lines 132-134).

**6. Review the number of samples evaluated, there is inconsistency in the number both in the text and in Table 2-Figure 1 (Study samples and sample size and result sections).**

We gathered 100 RCT positive COVID-19 samples; however, we had to exclude 01 of these samples because the date of symptom onset was unknown for this participant, making 99 the final pool of positive samples. We have made this correction in the paper as indicated (p. 8 line 205-206 and Table 1).

**7. I recommend that graph 1 become a table. Just specify the numeric data.**

We highly appreciate your recommendation and do understand how making it a table would save space and be more straightforward. However, we believe that the visualization of the confidence intervals in the graph make it easier and fast to convey the point of how the sensitivities vary according to each week, and this could potentially help the reader.

**8. Among the studies carried out in South America, there is a study in Ecuador of interest that could be added to the discussion "Diagnostic Performance of Seven Commercial COVID-19 Serology Tests Available in South America"**

We added the article you mentioned to the discussion, our results are in accordance of the findings of that study (p. 10, lines 261-264).

**Response to Reviewer 2:**

Thank you for your review of our paper. We have answered each of your points below.

**1. In the last paragraph of the background section it says:
“Our results contribute to the validation of these test brands to be used in seroprevalence studies and further evaluate its correlation with protective immunity or ability to detect vaccination/past infection status”

Overall, Covid-19 vaccines produce humoral immune responses to SARS-CoV-2 S protein, and antibodies to N protein appear due to infection, consequently, testing for protein-specific antibodies could be used to differentiate between different stages of the disease.
In this study, you don’t evaluate the correlation with protective immunity or the capacity of LFIA to detect vaccination or past infection status. Please, could you adjust this paragraph?**

Thank you for your comment. We completely understand what you mean, we meant to say that the validation of these brands is the first step for other next potential uses that will require exploring their correlation with protective immunity or ability to detect vaccination/past infection status. We have adjusted the paragraph you mentioned to try to convey what we meant (p. 5, lines 121-123).

**2. Could you clarify the first sentence of the last paragraph of the results section?
“Regarding the isotype target, the overall sensitivity was higher when incorporating either IgM or IG detection, followed by IgG and then IgM.”**

We meant to say that when considering a test positive when either IgM or IgG line is present, then sensitivity is higher, compared to when we considered a test positive when only IgM or only IgG is present. We have changed the wording to try to convey what we meant (p. 9, lines 233-240).

**3. Overall, the conclusions are a personal statement of your work and highlight the impact of the results, and future research that you considered needs to be done. So, What is the contribution of these references (32, 43–45) to the conclusions?
Maybe, it is more useful in the discussion section.**

We have followed your suggestions and removed the references from the conclusions and added them in the discussion section as suggested.

**4. Reference number 17 should be properly formatted**

We have formatted the reference as indicated.
